# PCA-Enhanced Deep Features for Alzheimer’s Disease Stage Classification with EFMM

**DOI:** 10.3390/diagnostics16152428

**Published:** 2026-07-31

**Authors:** Marwa Mawfaq Mohamedsheet Al-Hatab, Ruaa H. Ali Al-Mallah, Maysaloon Abed Qasim, Mohammed Falah Mohammed, Taha H. Rassem, Abdulghani Ali Ahmed

**Affiliations:** 1 Technical Engineering College, Northern Technical University, Mosul 41002, Iraq; marwa.alhatab@ntu.edu.iq; 2 Technical Engineering College for Computer and Artificial Intelligence, Northern Technical University, Mosul 41002, Iraq; ruaa_almallah@ntu.edu.iq (R.H.A.A.-M.); maysloon.alhashim@ntu.edu.iq (M.A.Q.); 3 Mechatronics Engineering Department, College of Engineering, University of Mosul, Mosul 41002, Iraq; mohammed.falah_kanna@uomosul.edu.iq; 4 School of Computer Science and Informatics, De Montfort University, Leicester LE1 9BH, UK; 5 School of Computer Science and Informatics, King’s College London, London WC2R 2LS, UK

**Keywords:** Alzheimer’s disease, magnetic resonance imaging, deep feature extraction, principal component analysis, machine learning, enhanced fuzzy min–max neural network, Alzheimer’s disease classification

## Abstract

**Background/Objectives**: Alzheimer’s disease (AD) is a progressive neurodegenerative disorder necessitating accurate and timely diagnosis for effective clinical intervention. While deep learning methods have shown promise in AD classification, many rely on computationally intensive architectures and high-dimensional feature representations. This study introduces a lightweight hybrid framework combining deep feature extraction, dimensionality reduction, and adaptive classification for MRI-based Alzheimer’s disease stage classification. **Methods**: Utilizing MRI images from a publicly available Alzheimer’s disease dataset encompassing four clinical stages (Non-Demented, Very Mild Demented, Mild Demented, and Moderate Demented), deep features were extracted using a pre-trained SqueezeNet model as a fixed feature extractor, generating 1000-dimensional feature vectors. Due to the computational complexity and for the improvement of the model efficiency, the dimensionality reduction technique, Principal Component Analysis (PCA) was then applied. This resulted in an optimum representation of 100 principal components, retaining about 96% of the variance. Then, the performances of various machine learning classifiers such as k-Nearest Neighbors (kNN), Support Vector Machine (SVM), Decision Tree (DT), Neural Network (NN), Naïve Bayes (NB), Logistic Regression (LR) and Enhanced Fuzzy Min–Max Neural Network (EFMM) were tested. The accuracy, precision, recall, F1-score, area under the receiver operating characteristic curve (AUC), and confusion matrices were used to evaluate the performance. Stratified 5-fold cross validation was used to ensure the strength of our results. **Results**: The findings show that PCA has a significant improvement in classification accuracy for most of the models. In particular, the EFMM classifier outperformed the other classifiers, with an accuracy of 97.19% on the independent test set. After PCA, the AUC values for classes such as Mild Demented, Moderate Demented, Non-Demented and Very Mild Demented were obtained as 97.12%, 99.97%, 93.79% and 95.26% respectively. We further validated our proposed framework using stratified 5-fold cross validation which further corroborated the robustness of our proposed framework. The EFMM achieved a mean accuracy of 98.38% ± 0.36 and a mean macro-F1 score of 98.48% ± 0.43. Friedman statistical testing demonstrated that there were significant differences between the performance of the classifiers evaluated (*p* < 0.001), which further validated the performance of the EFMM. **Conclusions**: To sum up, the proposed SqueezeNet–PCA–EFMM is an effective and efficient method for Alzheimer’s disease stage classification under MRI images. The combination of SqueezeNet, PCA, and EFMM—led not only to high classification performance, but also to good cross validation results. Furthermore, this property of incremental learning is the intrinsic one of the EFMM and renders this framework interesting for its incorporation in the next-generation intelligent clinical decision supports in particular, as medical care evolves.

## 1. Introduction

Alzheimer’s disease (AD) is the most common form of dementia, which is a loss of memory and cognitive function and a change in mood or personality [1]. It targets primarily the hippocampus, amygdala and other structures of the limbic system that play a key role in memory, emotions and other functions. cognitive function [2]. While many patients are initially found to have mild cognitive impairment (MCI), an intermediate state between normal cognition and AD, more severe symptoms develop later [3]. MCI is a major factor in the diagnosis of AD [4]. Diagnoses of AD can be made through a combination of clinical history, physical examinations, diagnostic tests, neurological examination, and the Mini-Mental State Examination (MMSE) [5,6]. But these heuristic tests are time consuming and may have inconsistent validity. AD specifically affects gray matter [7] and neuroimaging techniques serve to complement analysis of brain function in AD [8]. Artificial neural networks (ANNs), models of information processing based on artificial neurons, have demonstrated great potential for early diagnosis of AD. Using data from neuroimaging, ANNs can detect different patterns and signals related to the disorder [9,10]. Following sufficient training on a large collection of neuroimages, these networks can classify previously unseen data, which will greatly reduce the time taken for patients to manage and assist clinicians with the accuracy of the AD diagnosis and monitoring. The field of artificial intelligence (AI) has made great strides in areas such as natural language processing, computer vision, and autonomous systems. AI has also shown potential in medicine for diagnostics, imaging analysis and disease prediction, with its ability to make fast, accurate and repeatable evaluations from complex data [11,12,13]. Neural networks, fuzzy logic and hybrid models (e.g., fuzzy neural networks) [14,15] provide valuable contributions to the AD diagnostic toolbox. Many of the above methods solve the problems of traditional methods, such as objective, quantitative analysis of complex multimodal data, especially for imaging. Moreover, they are effective in managing uncertainty of diagnosis, facilitate early diagnosis and assist in personalized treatment strategies [13,16,17,18,19,20]. Even with data quality, interpretability, and clinical implementation issues persisting, AI shows significant promise in improving the precision, effectiveness, and promptness of Alzheimer’s diagnosis. This study was a feasibility study to assess a proposed classification framework based on MRI images of a publicly available Alzheimer’s disease dataset. Alzheimer’s stage images are categorized into four stages of Alzheimer’s disease: Non-Demented, Very Mild Demented, Mild Demented, and Moderate Demented [21]. These categories enable the evaluation of classification models in different stages of the disease and provide a useful reference for the evaluation of automated tools for Alzheimer’s staging. Although great progress has been made in learning for Alzheimer’s classification, there are still some practical problems. Deep convolutional neural networks have been used to achieve impressive results in medical image analysis, but they usually require huge amounts of annotated data, which may not be available in medical contexts [22]. Moreover, many deep learning models are black-box models, which are difficult to understand the reasons behind the prediction of individual models. This is especially crucial for clinical decision support in healthcare environments, where transparency, interpretability, and accountability are paramount [23].

Moreover, the dimensionality of the deep feature representations can lead to redundant representation of information, which could prompt the exploration of dimensionality reduction methods to build more compact and discriminative feature spaces. Previous works have reported encouraging results on classification performance based on deep learning models, but many fewer works have focused on incorporation of lightweight deep feature extraction, dimensionality reduction and adaptive fuzzy learning models for multi-stage classification of Alzheimer’s disease.

There have been many studies employing deep learning on the problem of Alzheimer’s disease classification; but few studies have focused on combining lightweight deep feature extraction with adaptive classification mechanisms. In this study, a hybrid approach is explored based on deep feature extraction, dimensionality reduction and adaptive machine learning. The pre-trained SqueezeNet model is chosen due to its lightweight architecture and computational efficiency and is used to extract deep representations. Principal Component Analysis (PCA) is then used to decrease the number of features to the most useful variance. The compact feature representation obtained is then tested with diverse machine learning classifiers and the Enhancement Fuzzy Min–Max Neural Network (EFMM) is highlighted for its superiority. Unlike other conventional machine learning classifiers EFMM uses adaptive fuzzy hyperboxes which are incrementally updated when fed with new samples. This property is especially appealing in medical decision support applications where the data set can change over time and retraining may be costly.

Furthermore, the hyperbox learning mechanism provides a balance between preserving previously acquired knowledge and incorporating new information, thereby addressing the well-known stability–plasticity dilemma that frequently arises in adaptive learning systems [14]. These characteristics motivated the investigation of EFMM as a lightweight and adaptive classification framework for Alzheimer’s disease stage classification. Although EFMM is not designed as an explicit explanation framework, its hyperbox-based representation provides a more structured description of decision regions than many conventional black-box deep learning classifiers [14]. The primary contributions of this paper are threefold: (1) the development of a hybrid Alzheimer’s disease classification framework combining SqueezeNet-based deep feature extraction, PCA-based dimensionality reduction, and EFMM classification; (2) a comprehensive comparative evaluation of EFMM against six widely used machine learning classifiers using multiple performance metrics, including accuracy, precision, recall, F1-score, and AUC; and (3) an investigation of the effectiveness of EFMM as an adaptive and computationally efficient classification framework for Alzheimer’s disease stage assessment under both hold-out and cross-validation evaluation protocols.

The remainder of this paper is organized as follows: Section 2 presents a brief review of the existing literature concerning the classification of Alzheimer’s disease through machine learning and deep learning methodologies. Section 3, titled Materials and Methods, delineates the dataset utilized, the preprocessing techniques employed, the process of feature extraction, the dimensionality reduction achieved via Principal Component Analysis (PCA), and the machine learning classifiers applied in this study. Section 4, Results and Discussion, presents the experimental findings in detail, comparing classifier performance and explaining the main outcomes and important observations. Finally, Section 5 concludes the study and suggests possible directions for future research.

## 2. Related Works

Recent advances in Alzheimer’s disease classification have increasingly relied on deep learning models to extract discriminative representations from neuroimaging data.

Sivera et al. modeled brain morphological changes associated with aging and Alzheimer’s disease using cross-sectional neuroimaging assessments [24]. Pan et al. subsequently demonstrated that mild cognitive impairment (MCI) could be predicted from FDG-PET images using a multi-view separable pyramid network that effectively captured disease-related patterns [25]. These studies demonstrated the potential of deep learning for neurodegenerative disease analysis. However, they generally relied on computationally intensive architectures, large annotated datasets, and substantial training resources.

CNN-based models have also been widely investigated for MRI-based Alzheimer’s disease classification. Tuvshinjargal and Hwang proposed a VGG-based transformation model with batch normalization for Alzheimer’s disease prediction using MRI data [26]. Lakhan et al. introduced an evolutionary deep convolutional neural network enabled by federated learning for Alzheimer’s disease detection [27]. Similarly, Sorour et al. demonstrated that deep features extracted using a lightweight pre-trained SqueezeNet model could achieve high classification performance for multi-stage Alzheimer’s disease classification [28]. These studies confirm the effectiveness of CNN-based feature learning. Nevertheless, most primarily focus on maximizing predictive accuracy while giving comparatively limited attention to feature compactness, computational efficiency, and adaptive learning capabilities.

More recently, researchers have explored advanced CNN- and Transformer-based architectures. Al Rahbani et al. integrated ResNet and EfficientNet with post-processing ensemble strategies and reported excellent classification performance on the ADNI and OASIS datasets [29]. Shaffi et al. investigated ensemble Vision Transformer architectures, including ViT, Swin Transformer, DeiT, and BEiT, under both data-rich and data-scarce scenarios [30]. Other recent studies have also explored Transformer-based models for learning spatial and sequential MRI representations [31,32]. Although these methods consistently achieve excellent classification performance, they generally rely on deeper architectures, ensemble strategies, or computationally expensive end-to-end training, which may limit their deployment in resource-constrained clinical environments. Hybrid frameworks that combine deep feature extraction with conventional machine learning classifiers have recently attracted increasing attention because they exploit the representational capability of deep neural networks while avoiding the computational cost associated with end-to-end model training. In particular, SqueezeNet-derived deep features have demonstrated promising performance for Alzheimer’s disease classification [28,33]. Nevertheless, existing studies have primarily focused on conventional classification models and have not comprehensively investigated the combined effect of lightweight deep feature extraction, PCA-based feature compression, and adaptive incremental fuzzy classification. Moreover, the use of classifiers capable of incremental learning has received comparatively little attention, despite their potential importance in evolving clinical environments where new patient data become available over time. Overall, the reviewed literature demonstrates that CNN- and Transformer-based methods can achieve excellent performance for Alzheimer’s disease classification. Nevertheless, several important challenges remain. First, many existing approaches rely on computationally demanding deep architectures. Second, high-dimensional deep feature representations often contain redundant information, increasing computational complexity and the risk of overfitting. Third, most existing classifiers are static and require complete retraining when new data become available. Finally, few studies have simultaneously investigated lightweight deep feature extraction, dimensionality reduction, and adaptive incremental classification within a unified framework. To address these limitations, the present study proposes a hybrid framework that combines lightweight SqueezeNet-based deep feature extraction, Principal Component Analysis (PCA) for feature compression, and the Enhanced Fuzzy Min–Max Neural Network (EFMM) for adaptive multi-stage Alzheimer’s disease classification.

## 3. Materials and Methods

### 3.1. Dataset

The data was provided in this study from the publicly available Augmented Alzheimer MRI Dataset on Kaggle [21]. The data set consists of 6400 magnetic resonance (MR) images split into four clinical phases: Non-Demented, Very Mild Demented, Mild Demented and Moderate Demented. The images are in JPEG format and are part of an existing public database of MRI images from people who have been diagnosed with Alzheimer’s disease. The problem is that the original set of images was quite imbalanced, so the creators created an augmented set of images to address this. Note that the data is not identified by subjects, does not contain patient metadata, acquisition site, or longitudinal follow-up data. This lack means that it is not possible to independently verify subject-level separation. Thus, the aim of this study is to assess image-level classification accuracy instead of the confirmed patient-level diagnostic accuracy. Stratified sampling was used in data partitioning to achieve an equal representation of all disease categories. This method ensured the same proportion of the class sizes in both the training and the testing sets. In particular, the total data set was split into 70% training and 30% testing subsets, with careful attention paid to the distribution of all four Alzheimer’s disease stages.

**Figure 1:**
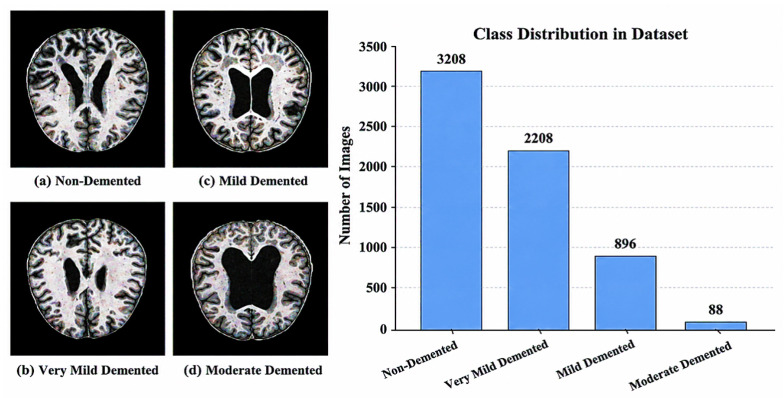
Representative MRI images and class distribution of the utilized Alzheimer’s disease dataset. (**a**) Mild Demented, (**b**) Moderate Demented (**c**) Non_Demented, (**d**) Very Mild Demented.

In addition to the primary 70:30 train–test partition, a stratified 5-fold cross-validation experiment was performed to evaluate model robustness under different data partitions. Each fold preserved the original class distribution to ensure fair evaluation across all Alzheimer’s disease categories. Classification performance was assessed using accuracy, precision, recall, F1-score, area under the receiver operating characteristic curve (AUC), confusion matrices, and cross-validation statistics. No additional data augmentation was performed by the authors. The study utilized the augmented MRI images provided within the publicly available Kaggle dataset. Consequently, all reported results reflect the characteristics of the dataset as distributed by the dataset creators. It should be noted that the Moderate Demented category contains substantially fewer samples than the other classes. Consequently, the classification performance reported for this class should be interpreted with caution, as the limited number of available samples may affect the generalizability of the results. Figure 1 presents representative MRI images from the four diagnostic categories together with the overall class distribution of the dataset

### 3.2. Experimental Environment

All experiments were conducted on a personal computer equipped with an Intel Core i5 processor and 16 GB of RAM. No GPU acceleration was used during feature extraction, dimensionality reduction, or classification. The pre-trained SqueezeNet model was employed as a fixed feature extractor, and activations from the global average pooling layer were used to generate 1000-dimensional deep feature vectors. Principal Component Analysis (PCA) was subsequently applied to reduce the feature space to 100 principal components prior to classification. The resulting feature representations were evaluated using the machine learning classifiers described in Section 3.6.

### 3.3. Deep Feature Extraction

Deep feature extraction was performed using a pre-trained SqueezeNet convolutional neural network. SqueezeNet was selected because of its lightweight architecture, reduced parameter count, and computational efficiency while maintaining competitive feature representation capability [34].

**Figure 2:**
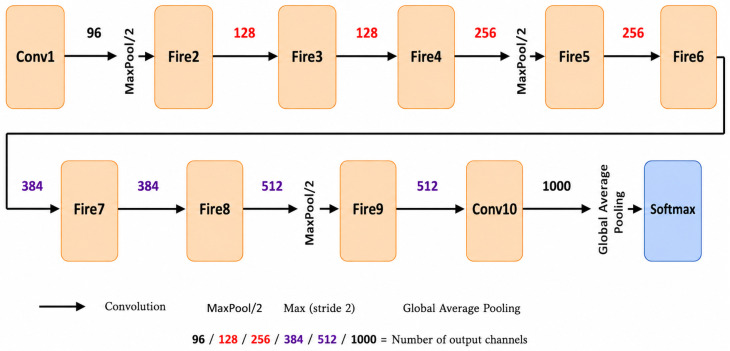
Simplified architecture of the pre-trained SqueezeNet model used for deep feature extraction. The numbers above the connections represent the output channels generated by each convolutional or Fire module, while MaxPool/2 indicates max pooling with a stride of 2. The activations from the global average pooling layer were extracted to generate a 1000-dimensional feature representation for each MRI image. No transfer learning or fine-tuning was performed.

The pre-trained network was used only as a fixed feature extractor in the present study. There is no transfer learning or fine-tuning of the network weights done. To obtain a deep feature vector, the activations from the global average pooling layer of each MRI image were propagated through the pre-trained network, resulting in a 1000-dimensional deep feature vector. These were passed as feature vectors to the next step of dimensionality reduction and classification. Figure 2 shows the simplified schematic architecture of SqueezeNet used in this study. The network is shown to be a series of Fire layers together with Max Pool layers followed by an initial convolutional layer and a global average pooling layer. This produced a 1000-dimensional feature vector, which was used for dimensionality reduction and classification and was used to capture high level characteristics of the images. Several PCA configurations have been tested in order to find a suitable compact representation; these are done by varying the number of retained principal components. The classification performance was tested for different configurations of the data, and finally the first 100 principal components were chosen as they gave an optimum classification performance while at the same time retaining about 96% of the total variance and reducing the dimensionality of the data.

### 3.4. Machine Learning Techniques

Machine learning classifiers were employed to evaluate the discriminative capability of deep feature representations extracted from MRI images and to assess the effect of Principal Component Analysis (PCA) on Alzheimer’s disease stage classification. Seven widely used classifiers, namely k-Nearest Neighbors (kNN), Naïve Bayes (NB), Logistic Regression (LR), Decision Tree (DT), Neural Network (NN), Support Vector Machine (SVM), and the Enhanced Fuzzy Min–Max Neural Network (EFMM), were evaluated using identical feature representations and experimental settings to enable a fair performance comparison. The subsequent subsections briefly summarize the principles of each classifier employed in this study.

The machine learning algorithms employed in this study are as follows:

#### 3.4.1. k-Nearest Neighbors (kNN)

The k-Nearest Neighbors (kNN) classifier operates by assigning a test sample to the class predominantly represented among its k nearest training samples, a determination made using the Euclidean distance metric as defined in Equation (1). This classifier is recognized for its simplicity, non-parametric nature, and its utility as an effective baseline for assessing the discriminative quality of extracted deep features.

(1) $$d\left(x,y\right)=\sqrt{\sum_{i=1}^{n}{\left(x_{i}-y_{i}\right)}^{2}}$$

where *x* and *y* represent feature vectors corresponding to two distinct data points, and *n* denotes the total number of features. The nearest neighbor for the given data point is identified based on the computed distances. The quantity of neighbors taken into account is dictated by a user-defined parameter, k [35].

#### 3.4.2. Naive Bayes (NB)

The Naïve Bayes (NB) classifier estimates posterior class probabilities by applying Bayes’ theorem under the assumption of conditional independence among features. Despite its inherent simplicity, NB offers a valuable probabilistic baseline for evaluating the efficacy of extracted feature representations [36]. Equation (2) formally describes the posterior probability estimation.

(2) $$P\left(A|B\right)=\frac{P(B|A)P(A)}{P(B)}$$

The probability of class (A, target) given the predictor (B, attributes), denoted as P, depends entirely on specific information or evidence [37].

#### 3.4.3. Logistic Regression (LR)

Logistic regression (LR) is a statistical method characterized by a set of rules that employs mathematical equations to delineate the relationship between input variables and a binary outcome. Central to LR is the logistic function, commonly referred to as the sigmoid function. This function transforms the output of a linear regression into a probability value constrained within the interval [0, 1]. The equation for the logistic function is as follows:

(3) $$P\left(X\right)=\frac{1}{1+e^{-z}}.$$

where P(X) represents the predicted probability of the positive class and *z* denotes the linear combination of the input features and their corresponding coefficients. The logistic transformation can also be expressed in log-odds form as:

(4) $$\log\left(\frac{P(x)}{1-P(x)}\right)=z$$

The difference in probability between the predicted and actual class labels is assessed using the cost function, often called log loss or cross-entropy loss. The objective during training is to minimize this cost function as much as possible. Below is the equation for the cost function:

(5) $$J\left(\theta \right)=-\frac{1}{N}\sum_{i=1}^{N}\left[y_{i}\log\left(P(X_{i})\right)+\left(1-y_{i}\right)\log\left(1-P(X_{i})\right)\right].$$

In the context of analysis, J(θ) denotes the cost function, while *N* signifies the large set of training examples. Here, $X_{i}$ represents the input elements for a specific training example, $y_{i}$ is the original class label for that example, and $P(X_{i})$ is the predicted probability for effective classification of that training example [38].

#### 3.4.4. Decision Trees (DT)

Decision Trees (DTs) recursively partition the feature space utilizing measures such as information gain and entropy to construct hierarchical decision rules. Their interpretable structure establishes them as a valuable benchmark for comparison with more sophisticated classifiers. Entropy is computed using Equation (6) [39].

(6) $$E=-\sum_{i=1}^{c}p\left(i\right)\log_{2}\left(p(i)\right)$$

where *c* represents the total number of classes, while p(i) indicates the proportion of samples in a node that belong to class *i*. Entropy values range from 0 to 1, with 0 signifying a perfectly pure node (where all samples belong to a single class) and 1 indicating maximum impurity (where samples are evenly distributed among all classes).

#### 3.4.5. Neural Networks (NN)

The Neural Network (NN) classifier consists of interconnected computational neurons that learn nonlinear decision boundaries through iterative optimization. Owing to their ability to model complex feature relationships, neural networks provide a robust baseline for comparison with the proposed EFMM classifier.

#### 3.4.6. Support Vector Machine (SVM)

Support Vector Machines (SVMs) identify an optimal separating hyperplane by maximizing the margin between distinct classes. Linear SVM classification is formally expressed by Equation (7), where the learned weights define the decision boundary within the feature space.

(7) $$f\left(x\right)=\sum_{i=1}^{n}w_{i}x_{i}+b$$

where *x* denotes the input feature vector, *w* represents the weight vector, and *b* is the bias term. The decision function f(x) determines the class assignment of a sample according to its position relative to the separating hyperplane. The hyperplane defined by f(x)=0 represents the decision boundary separating the classes. The margin is the distance between the hyperplane and the nearest factors [40].

### 3.5. Enhanced Fuzzy Min–Max Neural Network (EFMM)

The Enhanced Fuzzy Min–Max Neural Network (EFMM) is a hybrid neuro-fuzzy classifier that integrates adaptive learning capabilities of artificial neural networks with the interpretability of fuzzy set theory [14]. Distinct from conventional classifiers that necessitate complete retraining with the advent of new data, EFMM facilitates incremental learning by continuously adapting its decision regions without compromising previously acquired knowledge. This characteristic renders EFMM particularly well-suited for evolving medical datasets, where additional patient records may accumulate over time [41,42]. The foundational EFMM architecture and learning strategy are detailed in Mohammed and Lim [14].

#### 3.5.1. Hyperbox Representation

**Figure 3:**
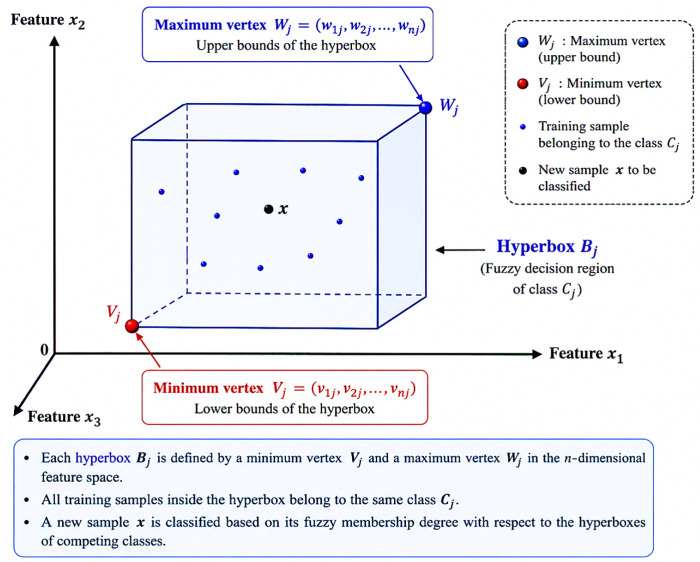
Geometric representation illustration of a hyperbox in the transformed feature space.

The EFMM classifier represents each class by one or more hyperboxes, which are multidimensional fuzzy regions delineated by a minimum and a maximum vertex. Each hyperbox encapsulates samples pertaining to the same class while accommodating gradual fuzzy membership near its boundaries. During the classification process, a sample’s membership value is determined by its relative position concerning these hyperbox boundaries. Figure 3 illustrates the geometric representation of a hyperbox in the transformed feature space.

In contrast to many black-box classifiers, hyperboxes offer an explicit geometric representation of the learned decision regions. Although this study does not focus on rule extraction or case-level explanation, the inherent hyperbox structure provides a transparent representation of the classifier, thereby establishing a foundation for future explainable clinical decision-support systems.

#### 3.5.2. EFMM Incremental Learning Procedure

**Figure 4:**
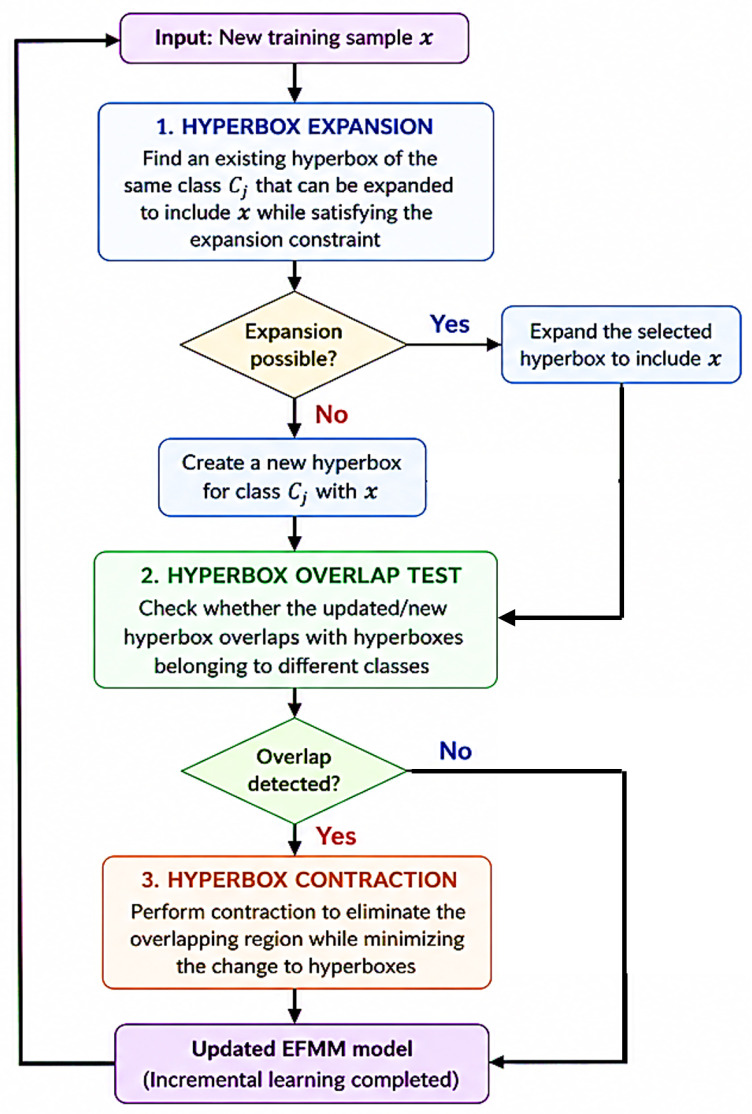
Overview of the EFMM learning procedure consisting of hyperbox expansion, overlap testing, and contraction during incremental learning

The EFMM model is based on incremental learning paradigm that consists of three successive learning steps [14]:

- Hyperbox Expansion: When a new training instance is introduced, the EFMM will first try to find a hyperbox that has been created for the class corresponding to the new instance. Once the new sample is incorporated into such a hyperbox, if it satisfies a predetermined size criterion, the hyperbox is then expand to include the new sample. Otherwise, a new hyperbox is created for the sample.
- Hyperbox Overlap Test: After a potential expansion of a hyperbox, the EFMM performs a critical overlap detection step. This means checking if the adjusted hyperbox overlaps with other hyperboxes of different classes. Identification and prevention of such overlaps is very important to keep clear decision boundaries and good class separability. If overlapped is detected between Hyperboxes from different classes, the EFMM performs a contraction process.
- Hyperbox Contraction: In instances where hyperbox overlap is detected, the EFMM performs a contraction operation. This process specifically designed to eliminate hyperboxes overlapped regions that belong to different classes. A key objective during contraction is to preserve the maximum possible amount of previously acquired knowledge, thereby maintaining classification accuracy without necessitating a retraining of the model. The entire learning process is summarized in Figure 4.

#### 3.5.3. Advantages for Alzheimer’s Disease Classification

The distributions of the deep features derived from MRI often overlap partially, especially between adjacent stages of Alzheimer’s disease (AD), e.g., Very Mild Demented and Mild Demented. This is an intrinsic problem that can be resolved by the Enhanced Fuzzy Neural Network Model (EFMM) which adaptively adjusts class boundaries in a systematic way by hyperbox expansion, overlap detection, and hyperbox contraction. The intrinsic mechanisms allow the classifier to acquire nonlinear and flexible decision regions and still retain its incremental learning capability. There are several unique benefits of EFMM for classification in comparison to conventional machine learning classifiers, which are directly related to classification [14]:

- Online Learning Capability: Enables data to be updated in real-time and be flexible, which is important for longitudinal clinical studies and streaming data applications.
- Structured Hyperbox Representation: EFMM uses adaptive fuzzy hyperboxes to represent classes, and provides a more structured representation of decision regions than most of the common black-box classifiers. In this study, no explicit explanation mechanisms exist, but there are future possibilities for interpretation analyses in the hyperbox structure.
- Imbalanced Data Class Competitive Performance: EFMM is based on adaptive hyperbox learning mechanism, which ensures competitive performance in imbalanced data classes. This property is especially important for the Alzheimer’s disease datasets, where the minority classes are often underrepresented.
- Adaptive Learning Capability: The main reason for choosing EFMM as the main classifier is its capability to learn incrementally and adapt boundaries through hyperbox expansion and contraction mechanisms.

The properties of EFMM are interesting for evolving datasets in medicine, in which new observations can be added to the data with time. Unlike many traditional classifiers, EFMM has the ability to continuously learn with the new samples by using adaptive hyperbox expansion and contraction. This ability enables the learning model to learn new things and keep the things it has learned, which reduces the stability–plasticity dilemma that is often encountered in adaptive learning systems. EFMM was investigated in the present study due to its aforementioned characteristics. With these features, EFMM is a potential classification tool for the stage assessment of Alzheimer’s disease. Its adaptive learning, efficient computation and structured hyperbox representation make it a potential for a very desirable intelligent clinical decision support system.

### 3.6. Principal Component Analysis

To reduce the dimensionality of the deep feature vectors extracted from SqueezeNet, Principal Component Analysis (PCA) was used. The first feature representation had 1000 features. The training data was used to fit the PCA model, and the testing data was used for the transformation with the parameters obtained from the training set, to prevent information leakage. This approach made sure that information in the test set did not affect the dimensionality-reduction procedure. PCA is a technique to transform the original feature space into a new set of orthogonal principal components, arranged in order of the variance explained [43]. Suppose that the original feature matrix is denoted by *X*. The transformed feature matrix *Z* is found as follows:

(8) Z=(X−μ)∗W

where: *X* = original feature matrix, μ = mean vector, and *W* = eigenvector matrix.

In this study, the original 1000-dimensional feature vectors were reduced to 100 principal components, preserving approximately 96% of the total variance while substantially reducing dimensionality and computational complexity.

**Figure 5:**
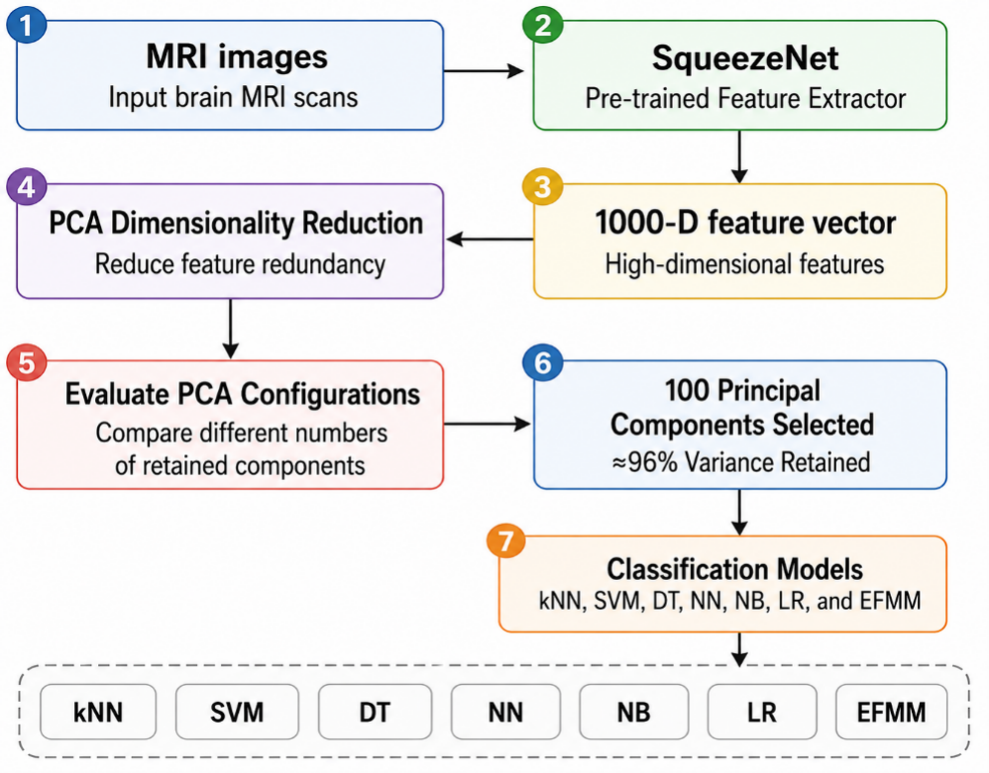
Proposed Alzheimer’s disease classification framework. MRI images are first processed using a pre-trained SqueezeNet model to extract 1000-dimensional deep features. PCA is then applied for dimensionality reduction, and different numbers of principal components are evaluated. The optimal representation consisting of 100 principal components is subsequently used for classification using seven machine learning models, including EFMM

Figure 5 illustrates the overall workflow of the proposed Alzheimer’s disease classification framework. MRI images are first processed using a pre-trained SqueezeNet network to extract 1000-dimensional deep feature vectors. Principal Component Analysis (PCA) is subsequently applied to reduce feature dimensionality. Different PCA configurations were experimentally evaluated, and the first 100 principal components were selected because they provided the best classification performance while preserving approximately 96% of the total variance. The resulting compact feature representation was then used as input to the evaluated machine learning classifiers, including kNN, SVM, DT, NN, NB, LR, and EFMM.

## 4. Results and Discussion

This study examines the utilization of machine learning (ML) models for predicting stages of Alzheimer’s disease (AD) based on processed MRI image data. The dataset comprises 6400 MRI images categorized into four distinct AD stages: Mild Demented, Moderate Demented, Non-Demented, and Very Mild Demented. A 70:30 train-test split was employed, with seventy percent of the data allocated for training and thirty percent for testing. Feature extraction was conducted using the pre-trained SqueezeNet network, resulting in 1000 features per image. Subsequently, Principal Component Analysis (PCA) was applied to reduce the dimensionality to 100 components while preserving 96% of the variance. Various ML models were assessed using key performance metrics to identify the most effective classifier.

### 4.1. Evaluation Metrics Before and After PCA

**Figure 6:**
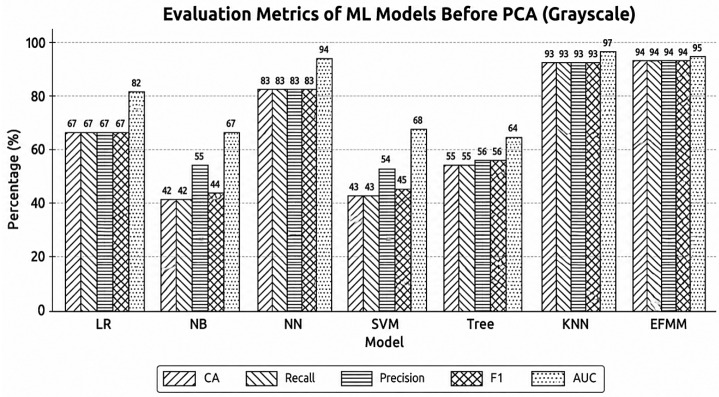
Evaluation Metrics of different ML Models Before the PCA.

**Figure 7:**
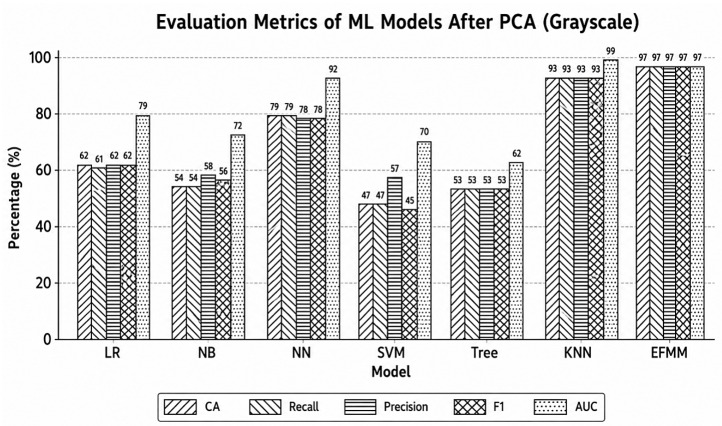
Evaluation Metrics of different ML Models After PCA.

The evaluation metrics employed to assess the performance of the models include Classification Accuracy (CA), Recall, Precision, F1 Score, and the Area Under the Receiver Operating Characteristic Curve (AUC). The performance results of the models, both prior to and following Principal Component Analysis (PCA), are presented in Table 1 and Table 2, respectively. Figure 6 provides a visual comparison of the evaluation metrics obtained by the different machine learning classifiers before PCA, while Figure 7 illustrates the corresponding performance after PCA-based dimensionality reduction. Among the classifiers analyzed, the Enhanced Fuzzy Min-Max Neural Network (EFMM) model exhibited superior overall performance. Prior to PCA, it achieved an accuracy of 94.17%, with precision and recall both at 94%, and an AUC of 95%. Following PCA, the performance of EFMM improved significantly, attaining an accuracy of 97.19%, precision and recall of 97%, and an AUC of 96.53%. This performance surpassed that of all other models tested, including the k-Nearest Neighbors (kNN) model, which, despite achieving a slightly higher AUC of 99% post-PCA, recorded inferior accuracy and F1-score.

As shown in Figure 6 and Figure 7, PCA improved the performance of most classifiers by reducing feature redundancy and retaining the most informative components. The improvement was particularly evident for EFMM, whose classification accuracy increased from 94.17% before PCA to 97.19% after PCA, demonstrating the effectiveness of the proposed PCA-enhanced feature representation.

The EFMM’s capacity to learn flexible decision boundaries through hyperbox expansion and contraction facilitated its adaptation to the complexities inherent in the Alzheimer’s Disease (AD) classification problem. Its interpretability and stability in classification across various stages of AD rendered it particularly effective. Other models demonstrated varying levels of performance; the decision tree achieved only 55% accuracy and an AUC of 64% prior to PCA, with further deterioration observed post-PCA. The Support Vector Machine (SVM) displayed moderate performance with an AUC of 68% but struggled with both recall and precision. Neural Networks (NN) reached an AUC of 94% and exhibited reasonable performance with an accuracy of 83%, while Naive Bayes (NB) and Logistic Regression (LR) yielded lower accuracy due to their underlying assumptions, such as feature independence.

### 4.2. Evaluation Confusion Matrix

**Figure 8:**
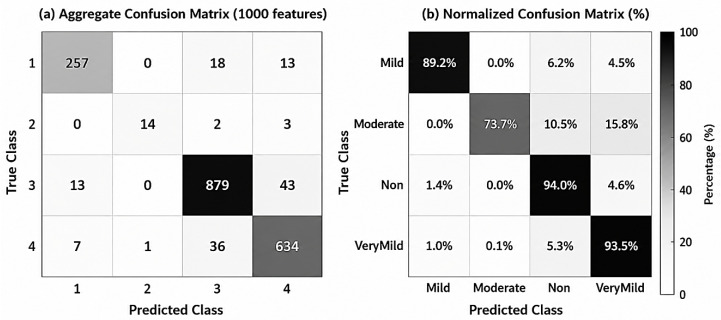
Aggregate and normalized confusion matrices for the EFMM model before PCA. Class 1 = Mild Demented, Class 2 = Moderate Demented, Class 3 = Non-Demented, and Class 4 = Very Mild Demented.

**Figure 9:**
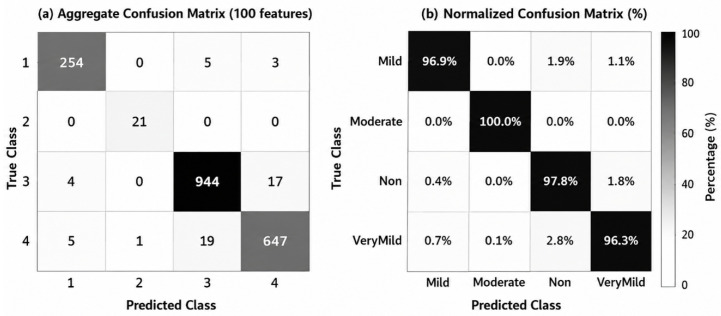
Aggregate and normalized confusion matrices for the EFMM model after PCA. Class 1 = Mild Demented, Class 2 = Moderate Demented, Class 3 = Non-Demented, and Class 4 = Very Mild Demented.

Figure 8 and Figure 9 present the confusion matrices for the EFMM model prior to and subsequent to the application of Principal Component Analysis (PCA). Prior to the implementation of PCA, the EFMM exhibited robust class-wise accuracy; however, there was notable confusion between the Mild and Very Mild Demented categories. Following the application of PCA, the model displayed enhanced precision and recall across all classes, as evidenced by an increase in diagonal dominance within the matrix, which signifies elevated true positive rates. Specifically:

- Mild Demented: 96.9% of samples were correctly classified.
- Moderate Demented: 100% of samples correctly classified.
- Non-Demented: 97.8% correct classification.
- Very Mild Demented: 96.3% correct classification.

This improvement substantiates that Principal Component Analysis (PCA) has augmented the model’s capacity to differentiate subtle distinctions among the early stages of Alzheimer’s disease (AD).

### 4.3. Evaluation of ROC Curves

**Figure 10:**
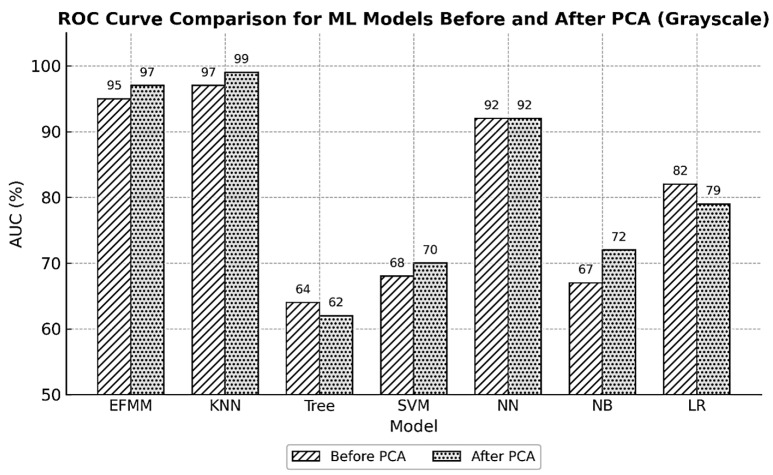
Comparison of multi-class one-vs-rest AUC values for the evaluated machine learning models before and after PCA.

Receiver Operating Characteristic (ROC) analysis assesses the efficacy of machine learning models in differentiating among the four stages of Alzheimer’s disease: Mild Demented, Moderate Demented, Non-Demented, and Very Mild Demented. Each stage is treated as a distinct target within a one-vs-rest framework. Figure 10 illustrates the ROC curves for the EFMM and benchmark models (k-Nearest Neighbors, Support Vector Machine, Neural Network, and Decision Tree) both prior to and subsequent to Principal Component Analysis (PCA). This comparative analysis elucidates the relative discriminative capabilities of each model across all operational thresholds.

EFMM exhibited robust ROC performance both prior to and following PCA, thereby affirming its capacity to effectively distinguish between classes with differing prevalence. Class-specific AUC metrics for EFMM are presented in Table 3 and Table 4.

Before PCA:

- Accuracy: 94.17%
- AUCs: Class 1–97.17%, Class 2–97.40%, Class 3–91.73%, Class 4–91.27%

After PCA:

- Accuracy: 97.19%
- AUCs: Class 1–97.12%, Class 2–99.97%, Class 3–93.79%, Class 4–95.26%

**Figure 11:**
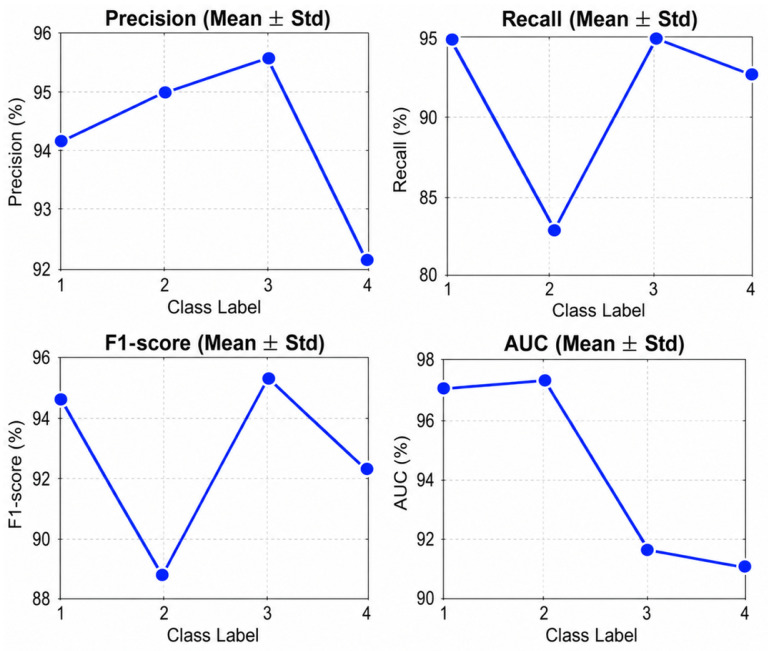
Summary of Class-wise Evaluation Metrics for the EFMM Before the PCA.

**Figure 12:**
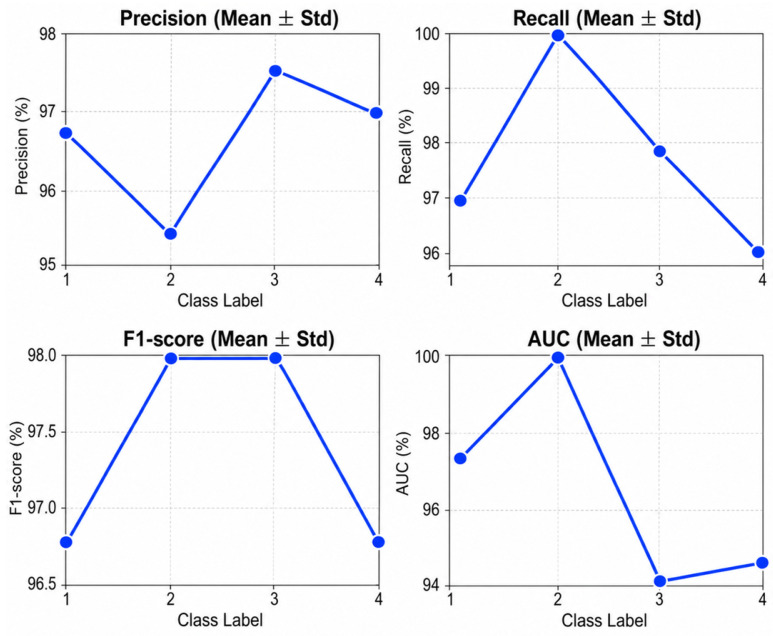
Summary of Class-wise Evaluation Metrics for the EFMM After the PCA.

Figure 11 and Figure 12 illustrate the class-wise performance of EFMM before and after PCA-based dimensionality reduction. Overall, PCA led to more balanced classification performance across the four Alzheimer’s disease stages, improving precision, recall, F1-score, and AUC for most classes. The most significant gains were observed in the Moderate Demented and Very Mild Demented categories, suggesting that PCA enhanced the discriminative capability of the extracted deep features by reducing redundancy and emphasizing the most informative components.

Key observations include:

- Mild Dementia (Class 1): The EFMM exhibited high sensitivity and precision, maintaining an area under the curve (AUC) near 97% both before and after principal component analysis (PCA).
- Moderate Dementia (Class 2): The EFMM significantly outperformed all other models following PCA, achieving near-perfect separation with an AUC of 99.97% and a recall of 100%.
- Non-Demented (Class 3): The EFMM demonstrated a slight improvement in AUC after PCA, increasing from 91.73% to 93.79%, while sustaining high levels of precision and recall.
- Very Mild Dementia (Class 4): The positive effect of PCA on the classification of early-stage dementia was observed with Very Mild Dementia (Class 4) with AUCs moving from 91.27% to 95.26%.

While KNN had the highest overall AUC value, EFMM consistently outperformed the other classifiers on the majority of the evaluation metrics, including the classification accuracy, F1 score and class-wise performance. Hence, EFMM achieved the best predictive performance and robustness among the classifiers that were tested. This separation emphasizes the idea that the AUC is a measure of the global ranking ability, whereas accuracy and F1 score are better suited to assessing the effectiveness of the classification at a given decision threshold, which is important in clinical applications. Based on these results, EFMM has the best overall performance among the classifiers evaluated under the experimental conditions used in this paper.

### 4.4. Evaluation Using 5-Fold Cross-Validation

The robustness and generalization ability of the Enhanced Fuzzy Min–Max Neural Network (EFMM) was further confirmed by performing 5-fold cross validation on the Alzheimer’s MRI dataset after deep feature extraction using SqueezeNet and dimensionality reduction using Principal Component Analysis (PCA). The data set was divided into five equal parts and each part was used as a test set while the other four parts were used as training sets. To ensure tight bounds on hyperbox sizes and promote accurate boundary formation, while reducing overfitting, a gamma value of 1 and a threshold parameter of Θ=0.1 were adopted.

Across the five folds, EFMM consistently attained high classification accuracy across all folds, ranging from 97.97% to 98.75%, with a mean accuracy of 98.38%±0.36 and a mean macro-F1 score of 98.48%±0.43. The low standard deviation observed across folds indicates that the proposed framework maintained stable performance under different training-testing partitions. Table 5 summarizes the fold-wise accuracies.

To determine whether the observed performance differences among classifiers were statistically significant, the Friedman non-parametric test was applied to the fold-wise results obtained from the stratified 5-fold cross-validation experiment. The Friedman test revealed statistically significant differences among the evaluated classifiers for both classification accuracy $\left(p=3.9\times 10^{-5}\right)$ and macro-$F_{1}$ score $\left(p=4.9\times 10^{-5}\right)$. These results indicate that the observed performance differences were unlikely to be attributable to random variation arising from the particular data partition used during evaluation.

These findings reinforce the ability of EFMM to generalize across different data partitions, complementing the hold-out evaluation presented in Section 4.1. Compared with the other machine learning models evaluated in this study, EFMM achieved the highest mean accuracy and macro-F1 score while simultaneously exhibiting the lowest performance variability across folds. Such consistency is particularly desirable for medical decision-support applications, where data distributions may vary across patient populations and acquisition conditions. The strong performance of EFMM may be attributed to its hyperbox-based representation, which can effectively model complex decision regions while accommodating class overlap. Furthermore, the incremental learning mechanism of EFMM allows adaptive refinement of class boundaries without requiring complete retraining. These characteristics may contribute to its robustness when handling high-dimensional deep feature representations reduced through PCA. The mean and standard deviation of the classification accuracy and macro-F1 score obtained across the five folds for all evaluated classifiers are summarized in Table 5.

### 4.5. Discussion Relative to Recent CNN- and Transformer-Based Alzheimer’s Disease Classification Studies

Table 6 summarizes several representative recent CNN- and transformer-based approaches for Alzheimer’s disease classification.

Recent advances in Alzheimer’s disease classification have been dominated by increasingly sophisticated deep learning architectures, including convolutional neural networks (CNNs) and Vision Transformers (ViTs). These approaches have demonstrated excellent classification performance by learning highly discriminative feature representations directly from neuroimaging data. For example, Al Rahbani et al. combined ResNet and EfficientNet with ensemble post-processing strategies and reported excellent performance on the ADNI and OASIS datasets [29]. Similarly, Shaffi et al. demonstrated that ensemble Vision Transformer architectures can achieve state-of-the-art classification accuracy under both data-rich and data-scarce conditions [30]. Other recent Transformer-based approaches have further improved performance by incorporating attention mechanisms and sequential modelling of MRI slices [31,32].

Although these methods have demonstrated remarkable predictive performance, they generally rely on computationally intensive architectures, large numbers of trainable parameters, and end-to-end optimization. Such models often require substantial computational resources and large annotated datasets for effective training, which may limit their deployment in clinical environments where computational resources or labelled data are limited. Moreover, most existing deep learning approaches employ static classifiers that require complete retraining whenever new training data become available.

In contrast, the proposed framework follows a hybrid strategy in which a lightweight pre-trained SqueezeNet model is employed exclusively for deep feature extraction, while Principal Component Analysis (PCA) is used to eliminate feature redundancy before classification using the Enhanced Fuzzy Min–Max Neural Network (EFMM). This design substantially reduces computational complexity because only the machine learning classifier is trained, while the deep feature extractor remains fixed. In addition, PCA compresses the original 1000-dimensional feature vectors into a compact 100-dimensional representation while preserving approximately 96% of the total variance, thereby improving computational efficiency without sacrificing discriminative information.

Another distinguishing characteristic of the proposed framework is the use of EFMM as the final classifier. Unlike conventional CNN or Transformer classifiers that typically employ a SoftMax output layer, EFMM supports adaptive incremental learning through hyperbox expansion and contraction mechanisms. Consequently, new observations can be incorporated without complete retraining of the classifier. Although this capability was not experimentally evaluated in an online learning scenario in the present study, it represents a potentially valuable characteristic for evolving clinical databases where new patient data become available over time.

From a predictive performance perspective, the proposed framework achieved 97.19% classification accuracy on the hold-out test set and 98.38% ± 0.36 under stratified five-fold cross-validation. These results are competitive with those reported by several recent CNN- and Transformer-based approaches while relying on a considerably simpler classification pipeline. Rather than attempting to outperform increasingly complex deep architectures, the objective of this work was to investigate whether lightweight deep feature extraction, dimensionality reduction, and adaptive fuzzy classification could provide an effective alternative for multi-stage Alzheimer’s disease classification. Nevertheless, the proposed framework has several limitations. The experiments were conducted using a single publicly available MRI dataset, and subject-level separation could not be independently verified because subject identifiers were unavailable. Consequently, the reported results represent image-level classification performance rather than confirmed patient-level diagnostic performance. Furthermore, external validation using independent datasets acquired from different institutions was not performed. Future work should therefore evaluate the proposed framework on multiple subject-level datasets and directly compare its performance with recent CNN- and Transformer-based methods under identical experimental protocols.

## 5. Conclusions

This study investigated the effect of Principal Component Analysis (PCA) on the classification of Alzheimer’s disease (AD) stages using deep features extracted from magnetic resonance imaging (MRI) data. A pre-trained SqueezeNet model was employed as a fixed feature extractor to generate 1000-dimensional feature representations, which were subsequently reduced using PCA and evaluated using multiple machine learning classifiers. The experimental results demonstrated that PCA substantially improved classification performance by reducing feature redundancy while preserving the most informative characteristics of the extracted deep features. Among the evaluated classifiers, the Enhanced Fuzzy Min–Max Neural Network (EFMM) achieved the highest overall classification performance, obtaining an accuracy of 97.19% on the hold-out test set and a mean accuracy of 98.38%±0.36 under stratified 5-fold cross-validation. In addition, Friedman statistical analysis confirmed significant performance differences among the evaluated classifiers (p<0.001). Although k-Nearest Neighbors (kNN) achieved slightly higher overall AUC values, EFMM consistently achieved higher accuracy, macro-F1 score, and cross-validation performance, indicating a favorable balance between predictive effectiveness and robustness. The strong performance of EFMM may be attributed to its adaptive hyperbox-based learning mechanism, which can effectively model complex decision boundaries while supporting incremental learning. Furthermore, PCA reduced the original 1000-dimensional feature vectors to 100 principal components while preserving approximately 96% of the total variance, resulting in a more compact and computationally efficient feature representation.

Several limitations should be acknowledged. First, the study was conducted using a single publicly available dataset, and subject-level separation could not be independently verified because subject identifiers were not provided. Consequently, although the proposed framework achieved high classification performance, the reported results should be interpreted as image-level classification performance rather than verified patient-level diagnostic performance. Second, the proposed framework was evaluated only on a single dataset, and external validation using independent datasets acquired from different institutions and imaging protocols was not performed. Therefore, although the proposed framework demonstrated encouraging performance, further validation using subject-level datasets from multiple clinical centers is necessary to establish its robustness, generalizability, and suitability for real-world clinical deployment.

In the future work, we will focus on evaluating the proposed framework using subject-level datasets collected from multiple institutions, performing external validation across different imaging protocols, and conducting direct comparisons with recent CNN- and Transformer-based architectures under identical experimental conditions. In addition, future research will investigate the integration of EFMM with explainable artificial intelligence techniques and incremental learning strategies to further enhance transparency and support intelligent clinical decision-making.

## Figures and Tables

**Table 1:** Evaluation Metrics for Different Machine Learning Models Prior to PCA. Precision, Recall, and F1-score represent macro-averaged values across the four Alzheimer’s disease classes. Multi-class AUC values were computed using a one-vs-rest strategy and averaged across classes.

Model	AUC (%)	F1 (%)	Precision (%)	Recall (%)	CA (%)
EFMM	95	**94**	**94**	**94**	**94.17**
KNN	**97**	93	93	93	93
DT	64	56	56	55	55
SVM	68	45	54	43	43
NN	94	83	83	83	83
NB	67	44	55	42	42
LR	82	67	67	67	67

Bold font indicates the highest values.

**Table 2:** Evaluation Metrics for Different Machine Learning Models After PCA. Precision, Recall, and F1-score represent macro-averaged values across the four Alzheimer’s disease classes. Multi-class AUC values were computed using a one-vs-rest strategy and averaged across classes.

Model	AUC (%)	F1 (%)	Precision (%)	Recall (%)	CA (%)
EFMM	96.53	**97**	**97**	**97**	**97.19**
KNN	**99**	93	93	93	93
Tree	62	53	53	53	53
SVM	70	45	57	47	47
NN	92	78	78	79	79
NB	72	56	58	54	54
LR	79	62	61	62	62

Bold font indicates the highest values.

**Table 3:** Class-wise Evaluation Metrics for the EFMM Before the PCA.

Class	Precision (%)	Recall (%)	F1 (%)	AUC (%)
1	94.1	95.42	94.76	97.17
2	95	82.61	88.37	97.40
3	95.58	94.98	95.28	91.73
4	92.15	92.84	92.49	91.27

**Table 4:** Class-wise Evaluation Metrics for the EFMM After the PCA.

Class	Precision (%)	Recall (%)	F1 (%)	AUC (%)
1	96.58	96.95	96.76	97.12
2	95.46	100	97.67	99.97
3	97.52	97.82	97.67	93.79
4	97	96.28	96.64	95.26

**Table 5:** Stratified 5-Fold Cross-Validation Performance of the Evaluated Classifiers After PCA-Based Dimensionality Reduction.

Model	Accuracy (%) Mean ± SD	Macro-$F_{1}$ (%) Mean ± SD
kNN	92.06±1.25	90.51±2.58
SVM	50.00±0.00	16.67±0.00
DT	52.39±1.20	37.78±1.78
NN	67.89±1.40	55.37±4.04
NB	62.34±1.48	54.16±4.70
LR	61.25±1.64	41.13±1.06
**EFMM**	**98.38 ± 0.36**	**98.48 ± 0.43**

Bold font indicates the highest values.

**Table 6:** Representative Recent CNN- and Transformer-Based Approaches for Alzheimer’s Disease Classification and Their Key Characteristics.

Study	Method	Dataset	Reported Performance	Key Characteristics
Al Rahbani et al. (2024) [29]	ResNet, EfficientNet, and post-processing ensemble	ADNI and OASIS MRI	ADNI: 98.59% EfficientNet, 94.59% ResNet, 98.97% post-processing. OASIS: 97.25% EfficientNet, 99.36% ResNet, 99.41% post-processing.	CNN-based multiclass AD classification using ensemble post-processing
Shaffi et al. (2024) [30]	Ensemble of Vision Transformers: ViT, Swin, DeiT, BEiT	OASIS and ADNI MRI	OASIS: individual VT accuracy 97.18–98.43%; best ensemble 99.29%. ADNI was also used to assess performance under data-scarce and imbalanced conditions.	Transformer ensemble for AD classification; compared against ML and CNN models
Alp et al. (2025) [31]	Vision Transformer + Time-Series Transformer	ADNI MRI	Balanced accuracy: 86.8%; F1-score: 86.6% under 10-fold cross-validation	Sequential MRI slice modeling using ViT features and time-series transformer
Jomeiri et al. (2025) [32]	Regional Attention-Enhanced Vision Transformer	sMRI	Accuracy: 94.2%; sensitivity: 91.8%; specificity: 95.7%; AUC: 0.96	Attention-enhanced transformer with regional biomarker focus
Qasim et al. (2026) [33]	SqueezeNet deep feature extraction + machine learning classification	MRI	Accuracy: 97.48%; sensitivity: 92.31%; specificity: 95.66%	Demonstrated the effectiveness of SqueezeNet-derived deep features for Alzheimer’s disease classification without PCA-based optimization or EFMM classification
This paper	SqueezeNet + PCA + EFMM	Kaggle MRI	97.19% hold-out accuracy; 98.38% ± 0.36 5-fold CV accuracy; 98.48% ± 0.43 macro-F1	Lightweight deep feature extraction with PCA compression and adaptive EFMM classification

## Data Availability

The data supporting the evaluation of the performance of the proposed methods in this study was collected from an online dataset, which can be accessed at the following link: Available online: https://www.kaggle.com/datasets/preetpalsingh25/alzheimers-dataset-4-class-of-images (accessed on 26 July 2025).
